# Polymorphisms of Vitamin D Signaling Pathway Genes and Calcium-Sensing Receptor Gene in respect to Survival of Hemodialysis Patients: A Prospective Observational Study

**DOI:** 10.1155/2016/2383216

**Published:** 2016-08-23

**Authors:** Alicja E. Grzegorzewska, Monika K. Świderska, Adrianna Mostowska, Wojciech Warchoł, Paweł P. Jagodziński

**Affiliations:** ^1^Chair and Department of Nephrology, Transplantology and Internal Diseases, Poznan University of Medical Sciences, Przybyszewskiego 49, 60-355 Poznań, Poland; ^2^Student Nephrology Research Group, Chair and Department of Nephrology, Transplantology and Internal Diseases, Poznan University of Medical Sciences, Przybyszewskiego 49, 60-355 Poznań, Poland; ^3^Chair and Department of Biochemistry and Molecular Biology, Poznan University of Medical Sciences, Święcickiego 6, 60-781 Poznań, Poland; ^4^Chair and Department of Biophysics, Poznan University of Medical Sciences, Grunwaldzka 6, 60-780 Poznań, Poland

## Abstract

We evaluated in the 7-year prospective study whether variants in vitamin D pathway genes and calcium-sensing receptor gene* (CASR)* are determinants of mortality in hemodialysis (HD) patients (*n* = 532). HRM analysis was used for* GC* rs2298849,* GC* rs1155563,* RXRA* rs10776909,* RXRA* rs10881578, and* CASR* rs7652589 genotyping.* GC* rs7041,* RXRA* rs749759,* VDR* rs2228570, and* VDR* rs1544410 were genotyped using PCR-RFLP analysis. The minor allele in* GC* rs2298849 was associated with all-cause mortality in univariate analysis (HR 1.330, 95% CI 1.046–1.692, *P* = 0.020). Bearers of the minor allele in* GC* rs2298849 demonstrated higher infection/neoplasm mortality than major allele homozygotes also in multivariate analysis (HR 2.116, 95% CI 1.096–4.087, *P* = 0.026). Cardiovascular mortality was associated with major homozygosity (CC) in* VDR* rs2228570 (HR 1.896, 95% CI 1.163–3.091, *P* = 0.010). CC genotype patients were more often dyslipidemic than TT genotype subjects (46.1% versus 31.9%, *P* = 0.047). Dyslipidemics showed higher frequency of rs1544410_rs2228570 haplotype AC than nondyslipidemics (26 versus 18%, *P*_corr_ = 0.005), whereas TT genotype patients were at lower risk of dyslipidemia compared with CC/CT genotype patients (HR 0.59, 95% CI 0.37–0.96, *P* = 0.04). In conclusion,* GC* rs2298849 and* VDR* rs2228570 SNPs are associated with survival on HD.* VDR*-related cardiovascular mortality may occur due to connections of rs2228570 with dyslipidemia.

## 1. Introduction

Patients who undergo renal replacement therapy (RRT) due to end-stage renal disease (ESRD) have higher prevalence of comorbidities and increased adjusted mortality risk compared with healthy population [1]. Cardiovascular diseases, infections, and cancers are the most common causes of death in this group of patients [1, 2].

Vitamin D (VD) deficiency is a mortality risk factor in hemodialysis (HD) patients [3, 4]. Associations between low VD status and neoplasms [5–7], diabetes mellitus [8, 9], cardiovascular disease [10], myocardial infarction [11], bone fractures [12], and others have been discovered in recent years. What is more, VD is an important immunoregulator [13] and might play a key role in susceptibility to autoimmune disorders [13–15]. Lower plasma concentrations of VD binding protein were also associated with increased mortality risk in a 4-year prospective follow-up of nondiabetic HD patients [16].

Polymorphisms of VD signaling pathway genes influencing VD status and proper VD utilization include group-specific component protein (VD binding protein) gene* (GC)*, VD receptor gene* (VDR),* and downstream mediator of VD signaling, retinoid X receptor alpha gene* (RXRA)* [17–19]. Calcium-sensing receptor, encoded by specific gene* (CASR)*, controls calcium homeostasis along with VD through the regulation of parathyroid hormone (PTH) production [20]. In secondary hyperparathyroidism, there is downregulation of vitamin D receptor, calcium-sensing receptor, and retinoid X receptor in parathyroid cells [21].

To date, few studies have investigated the effects of VD signaling pathway gene polymorphisms on survival of ESRD patients. Marco et al. [22] have found that BsmI polymorphism of* VDR* influenced survival of HD patients in a 4-year prospective study. However,* VDR* polymorphisms (BsmI and FokI) showed no impact on ninety-day survival rate of acute kidney injury patients [23].* GC*,* VDR*, and* RXRA* polymorphisms were not associated with risk of death in patients on RRT in our retrospective analysis [2].* CASR* polymorphic variants had no impact on all-cause and cardiovascular mortalities in HD patients as well as on the occurrence of cardiovascular events and all-cause mortality in renal transplant recipients in retrospective studies [24, 25]. As retrospective analyses have drawbacks, including biases in the selection of patients, long-term prospective studies may yield more promising results in evaluation of associations between mentioned polymorphisms and survival of HD patients, as seems to be confirmed by the Marco et al. study [22].

The aim of this 7-year prospective study was to evaluate whether single nucleotide polymorphisms (SNPs) of vitamin D signaling pathway genes and* CASR* are determinants of survival in HD patients.

## 2. Material and Methods 

### 2.1. Enrolment of Patients

HD patients living in the Greater Poland District, Poland, were enrolled into the prospective, observational study in January, 2009. Known status in respect to hepatitis B virus (HBV) susceptibility or infection was an inclusion criterion for our study, because the ability to produce antibodies to HBV surface antigen (anti-HBs) was one of parameters investigated as a predictor of survival. Therefore, the ability to produce anti-HBs antibodies had to be known at the start of the study. An exclusion criterion was renal transplantation prior to enrolment. Patients were in stable clinical condition for at least one month prior to enrolment. Characteristics of enrolled patients (*n* = 532) are shown in Table 1.

Coronary artery disease (CAD) was diagnosed based on a medical history, electrocardiograms, exercise stress test, and, in some cases, coronary angiography or computed tomography. Dyslipidemia was diagnosed according to the recommendations of the National Kidney Foundation/Kidney Disease Outcomes Quality Initiative (KDOQI) clinical practice guidelines [26].

### 2.2. Follow-Up

Patients were followed from January 30, 2009, to January 30, 2016. Results concerning 6-year survival (to January 30, 2015) of the same group of patients in respect to their ability to produce anti-HBs antibodies are presented in our earlier study [27].

The immediate cause of death was reported by a physician who declared death basing on medical evidence prior to death. Patients were mainly dying at hospital, and a nephrologist responsible for dialysis was usually arranging a patient hospitalization. Deaths that occurred outside medical facilities (at home, during outdoor activities) were declared by first aid physicians. Medical records of a deceased patient were available in each such case. The causes of death were recorded and categorized as cardiac (reported as myocardial infarction, sudden cardiac death, severe arrhythmias, cardiomyopathies, or cardiac failure), vascular (reported as cerebrovascular events, cerebral stroke, or generalized atherosclerosis), infectious (reported as sepsis, pneumonia, limb necrosis, pyonephrosis, or acute abdomen with peritonitis), neoplasms, or other/unknown.

Characteristics of HD patients at the end of a 7-year prospective study are shown in Table 2.

### 2.3. Genotyping

Genotyping of vitamin D signaling pathway genes* (VDR* rs2228570,* VDR* rs1544410,* RXRA* rs10776909,* RXRA* rs10881578,* RXRA* rs749759,* GC* rs2298849,* GC* rs7041, and* GC* rs1155563) was performed as previously described [2, 24, 28–31].

In brief, genomic DNA for a genotype analysis was isolated from peripheral blood lymphocytes by a salt-out extraction procedure. High-resolution melting curve (HRM) analysis was used for* GC* rs2298849,* GC* rs1155563,* RXRA* rs10776909,* RXRA* rs10881578, and* CASR* rs7652589 genotyping.* GC* rs7041,* RXRA* rs749759,* VDR* rs2228570, and* VDR* rs1544410 were genotyped using the polymerase chain reaction-restriction fragment length polymorphism (PCR-RFLP) analysis. The characteristics of analyzed polymorphisms are described in Supplementary Table 1 in Supplementary Material available online at http://dx.doi.org/10.1155/2016/2383216. Primer sequences and conditions for PCR-RFLP and HRM analyses are presented in Supplementary Table 2. Approximately 10% of the randomly chosen samples were regenotyped. Samples that failed the genotyping were excluded from further statistical analyses.

Genotyping of tested SNPs was performed in groups of 435–472 patients (Table 3).

### 2.4. Statistical Methods

The results are presented as numbers and percentages for categorical variables. Medians and ranges for continuous variables are shown as data sets were nonnormally distributed by the Shapiro-Wilk test in the majority of subgroups.

The Hardy-Weinberg equilibrium (HWE) was analyzed to compare the observed genotype frequencies to the expected ones using the Chi-square test (*P* > 0.01 with df = 1 for equilibrium).

Survival analyses were conducted using the Kaplan-Meier method with the log rank test or with calculation of multiple *P* value when more than two groups were compared. The Cox proportional hazard model was applied to show whether and to what extend the effect of a unit increase in a covariate was multiplicative with respect to the hazard rate of death.

Cox proportional hazard model was also applied in multivariate analyses assessing the contribution of demographics and clinical measures to mortality.

Abovementioned statistical analyses were performed using Graph-Pad InStat 3.10, 32 bits for Windows (GraphPad Software, Inc., San Diego, California, United States) and Statistica version 12 (Stat Soft, Inc., Tulsa, Oklahoma, United States).

Haplotype frequencies were estimated using the Haploview 4.2 software (http://www.broad.mit.edu/mpg/haploview/). Statistical significance was assessed using the 1000-fold permutation test.

Epistatic interactions were analyzed using the logistic regression and epistasis option in the PLINK software (http://pngu.mgh.harvard.edu/purcell/plink/).

A *P* value of less than 0.05 was considered significant. Borderline significance was defined as a *P* value between 0.05 and 0.10.

### 2.5. Ethics Approval of Research

The study design was approved by the Institutional Review Board of Poznan University of Medical Sciences, Poland. The written informed consent was obtained from all study participants.

## 3. Results

At the beginning of the study, distributions of tested polymorphisms were consistent with HWE.

### 3.1. The Kaplan-Meier Survival Analyses

Association of demographic, clinical, and laboratory characteristics of HD patients at enrolment with their all-cause mortality during the 7-year follow-up is demonstrated in Table 1. Longer survival was attributed to chronic glomerulonephritis (HR 0.675, 95% CI 0.488–0.935, *P* = 0.018) and polycystic kidney disease (HR 0.521, 95% CI 0.292–0.927, *P* = 0.027) as causes of ESRD, and the ability to develop antibodies to HBV surface antigen in response to HBV vaccination or infection (HR 0.649, 95% CI 0.478–0.800, *P* = 0.005). Shorter survival was demonstrated in patients with older age at the beginning of the study (HR 1.027, 95% CI 1.018–1.035 per each 1-year increase, *P* < 0.000001), CAD (HR 2.101, 95% CI 1.682–2.626, *P* < 0.000001), diabetic nephropathy (HR 1.577, 95% CI 1.248–1.994, *P* = 0.0001), and lower serum PTH concentrations (HR 1.035, 95% CI 1.011–1.059 per each 100 pg/mL decrease, *P* = 0.004).

**Figure 1:**
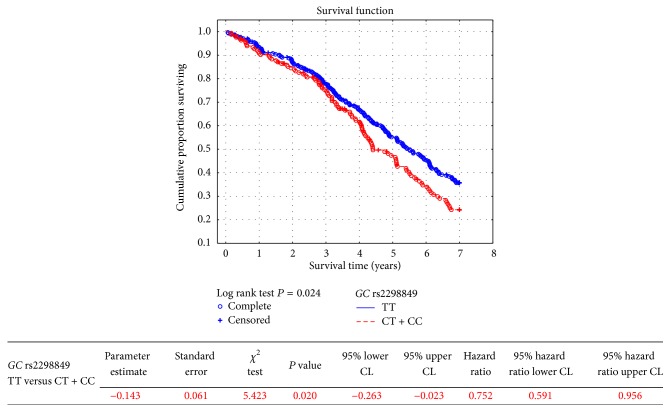
The probability of survival in hemodialysis patients in respect to* GC* rs2298849 polymorphic variant.

**Figure 2:**
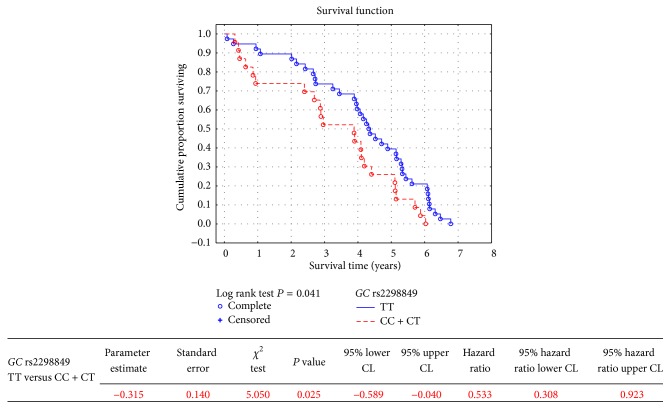
Infection-related and neoplasm-related mortality in hemodialysis patients in respect to* GC* rs2298849 polymorphic variant.

Among tested polymorphisms, only* GC* rs2298849 was significantly associated with all-cause mortality of HD patients (Table 3). Homozygotes of major allele showed significantly lower risk of death compared to patients bearing the minor allele in* GC* rs2298849 (HR 0.75, 95% CI 0.59–0.96, *P* = 0.020, Figure 1). In the next step, we tried to show which of the main causes of death (cardiovascular, infection-related, or neoplasm-related) were the most close to statistical significance being revealed for all-cause mortality. Log rank test, applied as the first-line evaluation, did not show significant *P* values for cardiovascular (Table 4), infection-related (Supplementary Table 3), and neoplasm-related (Supplementary Table 4) mortalities in respect to* GC* rs2298849 polymorphic variants. The HRs with 95% CI were, respectively, 0.96, 0.71–1.30, *P* = 0.796 (Supplementary Figure 1), 0.41, 0.22–0.96, *P* = 0.040 (Supplementary Figure 2), and 0.64, 0.28–1.47, *P* = 0.291 (Supplementary Figure 3) for these causes of death analyzed between major allele homozygotes and patients bearing the minor allele. Therefore, the association of* GC* rs2298849 with cardiovascular mortality could be excluded (Supplementary Figure 1). When noncardiovascular causes of death (infection-related, neoplasm-related, others beyond cardiovascular) were analyzed together in respect to in* GC* rs2298849 polymorphism, no significance was revealed between major allele homozygotes and patients bearing the minor allele (*P* value of 0.252 for log rank test, HR 0.79, 95% CI 0.53–1.18, *P* = 0.243). When infection-related and neoplasm-related mortalities were analyzed together, their significant association with* GC* rs2298849 was shown in the Kaplan-Meier analysis (log rank test *P* = 0.041) and in the Cox model (HR 0.53, 95% CI 0.31–0.92, *P* = 0.025), indicating better survival in homozygotes of major allele (Figure 2). 

**Figure 3:**
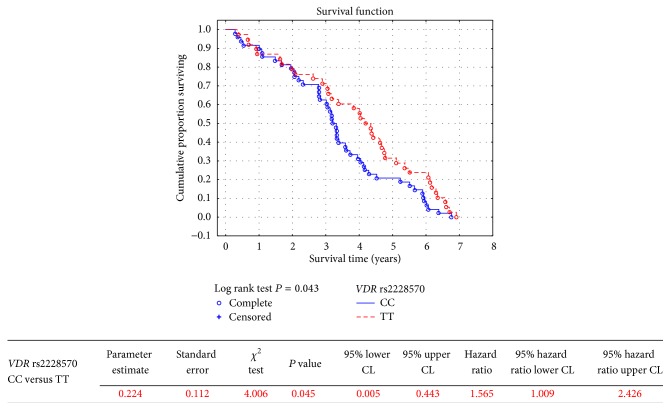
Cardiovascular mortality in hemodialysis patients in respect to* VDR* rs2228570 polymorphic variant.

Cardiovascular mortality was associated with* VDR* rs2228570 polymorphism in additive model of inheritance (Table 4). Homozygotes of the major allele showed significantly higher risk of cardiovascular death compared to homozygotes of the minor allele (HR 1.565, 95% CI 1.009–2.426, *P* = 0.045, Figure 3).

There were no significant associations between infection-related (Supplementary Table 3) or neoplasm-related (Supplementary Table 4) mortalities and tested polymorphisms except those already mentioned above for* GC* rs2298849.* RXRA* rs10776909 and* CASR* rs7652589 yielded a borderline significance in respect to infection-related mortality in the dominant model of inheritance (Supplementary Table 3).

### 3.2. Multivariate Analyses of Survival

Variables that yielded significance in univariate analyses of survival (age at the beginning of the study, diabetic nephropathy, polycystic kidney disease and chronic glomerulonephritis as causes of ESRD, CAD, the ability to develop antibodies to HBV surface antigen in response to HBV vaccination or infection, and serum PTH concentration) as well as RRT vintage prior to the study onset were used in multivariate analyses together with each polymorphic variant being also solely shown as involved in survival (*GC* rs2298849 and* VDR* rs2228570).

In the Cox model including 8 abovementioned variables and* GC* rs2298849, significant predictors of 7-year all-cause mortality were age at the beginning of the study (HR 1.016, 95% CI 1.006–1.026 per each 1-year increase, *P* = 0.006), RRT vintage prior to the study onset (HR 1.055, 95% CI 1.017–1.094 per each 1-year increase, *P* = 0.004), serum PTH concentration (HR 1.032, 95% CI 1.004–1.060 per each 100 pg/mL decrease, *P* = 0.004), CAD (HR 1.653, 95% CI 1.285–2.127, *P* = 0.00009), and the inability to develop antibodies to HBV surface antigen in response to HBV vaccination or infection (HR 1.494, 95% CI 1.090–2.048, *P* = 0.013). Among these variables, the minor allele in* GC* rs2298849 showed only borderline association with all-cause mortality since the study beginning (HR 1.253, 95% CI 0.982–1.599, *P* = 0.069), maybe due to the fact that patients bearing the minor allele in GC rs2298849 showed longer RRT vintage prior to the study onset compared to homozygotes of the major allele (2.6, 0.0–24.7 years versus 2.0, 0.0–22.2 years, *P* = 0.023) (Supplementary Table 5). However, the minor allele in* GC* rs2298849 was found as the significant independent predictor of together analyzed infection-related and neoplasm-related mortalities (HR 2.116, 95% CI 1.096–4.087, *P* = 0.026) among the following variables: age at the beginning of the study (HR 0.959, 95% CI 0.926–0.994 per each 1-year increase, *P* = 0.021), diabetic nephropathy (HR 2.061, 95% CI 1.012–4.195, *P* = 0.046), serum PTH concentration (HR 1.088, 95% CI 1.021–1.159 per each 100 pg/mL decrease, *P* = 0.009), and the inability to develop antibodies to HBV surface antigen in response to HBV vaccination or infection (HR 6.204, 95% CI 2.388–16.119, *P* = 0.0002).

In multivariate analysis, cardiovascular mortality in HD patients was associated with CAD (HR 1.671, 95% CI 1.028–2.718, *P* = 0.038), diabetic nephropathy (HR 1.825, 95% CI 1.094–3.043, *P* = 0.021), and major homozygosity (the CC genotype) in* VDR* rs2228570 (HR 1.896, 95% CI 1.163–3.091, *P* = 0.010). When death rates due to specific cardiovascular diseases were analyzed in respect to* VDR* rs2228570 polymorphic variants, the CC genotype was associated with higher risk of sudden cardiac death (OR 2.01, 95% CI 1.09–3.75, *P* = 0.024). HD patients, who had sudden cardiac death (*n* = 20), showed dyslipidemia in 55% and CAD in 50% of cases.

Patients harboring the CC genotype in* VDR* rs2228570 were more often dyslipidemic compared to subjects showing minor homozygosity (the TT genotype) (46.1% versus 31.9%, *P* = 0.047) (Supplementary Table 6). However, dyslipidemia in HD patients was not associated with frequency of major homozygosity in* VDR* rs2228570. Among all HD subjects tested for* VDR* rs2228570, dyslipidemic and nondyslipidemic patients did not differ significantly in prevalence of the CC genotype (31.6% versus 26.3%, *P* = 0.271). There was a trend for lower TT genotype frequency in dyslipidemic than in nondyslipidemic patients (16.0% versus 24.4%, *P* = 0.042). Patients showing the TT genotype were at lower risk of dyslipidemia compared to patients with CC and CT genotypes (HR 0.591, 95% CI 0.365–0.957, *P* = 0.042).

### 3.3. Haplotype Frequencies and Epistatic Interactions

Analyses of haplotype frequencies (Supplementary Tables 7 and 8) and epistatic interactions between tested genes (Supplementary Tables 9 and 10) did not reveal significant results in patients who died on HD and those who survived on HD for 7 years as well as in patients who died on HD due to cardiovascular diseases and those who survived on HD for 7 years.

Dyslipidemic HD patients showed higher frequency of the* VDR* rs1544410_rs2228570 haplotype AC than nondyslipidemic subjects (26% versus 18%, *P*_corr_ = 0.005) (Supplementary Table 11). A risk of dyslipidemia was 1.6-fold higher in subjects showing this haplotype (OR 1.640, 95% CI 1.151–2.338, *P* = 0.006).* VDR* rs1544410 and* VDR* rs2228570 showed also a significant epistatic interaction (FDR-adjusted *P* = 0.027) (Supplementary Table 12).

## 4. Discussion

VD deficiency [3, 4] and lower plasma concentrations of VD binding protein [16] were already associated with increased mortality risk in HD patients.* VDR* rs1544410 was shown as influencing survival of Catalonian HD patients in a 4-year prospective study [22]. Our 7-year prospective study on Greater Poland HD subjects did not confirm results of this previous study in respect to* VDR* rs1544410 polymorphism. We have revealed that* GC* rs2298849 polymorphic variant showed association with together analyzed infection-related and neoplasm-related causes mortality, whereas* VDR* rs2228570 was associated with cardiovascular mortality in prevalence HD patients.

In our retrospective studies, there were no associations between* GC* rs2298849 and age at RRT onset [32], gender [33], and survival evaluated either since the start of RRT or since birth [2].* GC* rs2298849 was also not associated with chronic glomerulonephritis [29] or type 2 diabetic nephropathy [30] as causes of ESRD. CAD in HD patients dialyzed due to type 2 diabetic nephropathy was not found to be associated with* GC* rs2298849 [30] and a response to HBV vaccination was not influenced by* GC* rs2298849 [28]. In this prospective study, the minor allele in* GC* rs2298849 was recognized as the risk allele for shorter survival on regular HD therapy. The Linkage Disequilibrium (LD) plot of HapMap SNPs within the* GC* region showed that the rs2298849 variant, located within the second LD block, was in perfect LD (*r* ^2^ = 1) with the intronic variant, rs1352845 [31]. However, this variant was up today associated only with total hip bone mineral density in postmenopausal Thai women [34]. We could not find associations between* GC* rs2298849 and phenotypes characterizing HD groups categorized by* GC* rs2298849 genotypes. However, the present study indicates that associations could be searched among infection and neoplasm diseases. The Kaplan-Meier survival curves demonstrate higher infection-cause and neoplasm-cause mortalities in HD patients bearing the C allele in* GC* rs2298849; however, the independent association of this allele with both mentioned causes of death was not shown in statistical analyses, maybe due to a relatively small number of HD patients dying from infections or neoplasms. As the C allele in* GC* rs2298849 seemed to be associated with increased mortality due to infections as well as due to neoplasms, a combined analysis, increasing a number of analyzed patients, yielded statistical significance. Recent data show that* GC* rs2298849 may be involved in the risk of ovarian cancer among noncarriers of BRCA1/BRCA2 mutations [35]. There is also a possibility that* GC* rs2298849 may be associated with infections or neoplasms through its influence on VD status. It is documented that VD deficiency contributes to neoplasm disease [5–7] and infections [36–38]. On the other hand, data on* GC* rs2298849 in association with VD status are controversial [31, 39–41]. Larger studies evaluating VD status, VD associated genes, and specific causes of mortality are needed to show independent associations between tested variables.

*GC* rs2298849, significantly associated with all-cause mortality in univariate analysis, remained only on borderline level of significance in the Cox multivariate analysis. As our study was performed on prevalent HD patients, bearers of the minor allele in* GC* rs2298849 appeared to be approximately 6 months longer on RRT prior to the study than homozygotes of the major allele. The study on incidence HD patients could be helpful in avoiding this possible confounding variable.

In Han Chinese adults with normal renal function, the minor allele in* VDR* rs2228570 was associated with CAD, whereas the major allele homozygosity in* VDR* rs2228570 was connected with higher plasma HDL-cholesterol concentrations in CAD patients [42]. Our study showed that the major allele homozygosity in* VDR* rs2228570 polymorphism was a predictor of cardiovascular mortality in prevalent HD patients. Our study also revealed that* VDR* rs1544410 and* VDR* rs2228570 are in epistatic interaction, and the* VDR* rs1544410_rs2228570 haplotype AC is associated with dyslipidemia in HD patients. Interestingly, López-Mejías et al. [43] recently demonstrated an association of* VDR* GATG haplotype with atherosclerotic disease in rheumatoid arthritis. The allele T of this haplotype is the minor allele in* VDR* rs1544410 and the allele G of this haplotype denotes the major allele in* VDR* rs2228570. This finding is in logical concordance with our observation showing more dyslipidemic patients among the group presenting the major allele homozygosity in* VDR* rs2228570 or the* VDR* rs1544410_rs2228570 haplotype AC. Dyslipidemia, occurring in 46.1% of the major homozygotes in* VDR* rs2228570, could contribute to generalized atherosclerosis, CAD, cerebral events, and finally to death. However, death rates due to myocardial infarction or cerebral stroke were not related to the major allele homozygosity in* VDR* rs2228570. On the other hand, sudden cardiac death, which was shown to be associated with the CC genotype in our study, was also attributed to CAD [44] and abnormal plasma lipids [45], both present in at least 50% of affected patients.

We were not able to show direct associations of tested polymorphisms with infection-related mortality. However,* GC* rs2298849,* RXRA* rs10776909, and* CASR* rs7652589 yielded a borderline significance in the dominant model of inheritance. In our recent study [24], there was an epistatic interaction between* CASR* rs7652589 and rs1024611 in the chemokine (C-C motif) ligand 2 gene (*CCL2*) in nephrolithiasis-related ESRD. The promoter polymorphism of* CCL2* was involved in viral [46] and bacterial [47] infections. In this study, 39 patients (7.3%) died due to infection. Larger group of studied patients or prolongation of the study for further years may clarify this borderline findings.

Neoplasm-related mortality occurred in 5.6% HD patients and was caused by cancers of various organs (gastrointestinal tract, kidney, bone marrow, and others). This diversity together with low number of affected patients could be a reason why we did not show any associations of tested polymorphisms with a mortality risk due to neoplasms, although such connections were described [35, 48, 49].

Mortality rate in HD patients in still high. In the study by Buargub [50], 51.4% patients expired during the 5-year follow-up. In this study, at least 59.6% patients died during 7-year period (outcomes of 66 transplant recipients and 7 patients moving to other centers are not included). Therefore, to recognize factors associated with mortality in this group is of great importance. Knowledge of appearance of risk polymorphisms in HD subjects may be helpful in choosing therapeutic strategies. It might be a challenge for the future to find out what outcome after renal transplantation occurs in patients showing the risk polymorphisms in* GC* rs2298849 or* VDR* rs2228570.

### 4.1. Study Limitations

Circulating 25(OH)D was determined in only 70 patients at the start of the study; therefore sample power was too small for analyses of survival (Supplementary Figure 4). However, determination of total and free vitamin D could be relevant, particularly that serum PTH concentration was among independent predictors of 7-year survival in the examined HD patients.

## 5. Conclusions

1. *GC* rs2298849 polymorphic variant is independently associated with infection/neoplasm mortality, whereas* VDR* rs2228570 is associated with cardiovascular mortality in prevalence HD patients.
2. Dyslipidemia occurs more frequently in the major than in the minor homozygotes of* VDR* rs2228570, and major homozygosity contributes to increased risk of cardiovascular mortality.
3. The* VDR* rs1544410_rs2228570 haplotype AC is associated with dyslipidemia in HD patients, whereas the TT genotype in* VDR* rs2228570 seems to have a protective role against dyslipidemia.

## Supplementary Material

The Supplementary Material contains characteristics of the analyzed polymorphisms and conditions for their identification (*Supplementary Tables 1-2*), associations of vitamin D signaling pathway genes and *CASR* with infection-related and neoplasm-related mortalities (*Supplementary Tables 3-4*), characteristics of patients in respect to *GC *rs2298849 and *VDR *rs2228570 polymorphic variants (*Supplementary Tables 5-6*), haplotype frequencies in patients who died on HD and those who survived on HD for 7 years in respect to all-cause and cardiovascular mortality (*Supplementary Tables 7-8*), epistatic interactions between genes in patients who died on HD and those who survived on HD for 7 years in respect to all-cause and cardiovascular mortality (*Supplementary Tables 9-10*), and haplotype frequencies and epistatic interactionsbetweengenes in dyslipidemic HD patients and non-dyslipidemic HD subjects(*Supplementary Table 11-12*). Cumulative proportion surviving curves are presented for cardiovascular, infection-related and neoplasm-related mortalities in hemodialysis patients in respect to *GC *rs2298849 polymorphism (*Supplementary Figures 1-3*) as well as in respect to circulating 25(OH)D at the start of the prospective study (*Supplementary Figure 4*).

## Figures and Tables

**Table 1:** Demographic, clinical, and laboratory characteristics of hemodialysis patients at enrolment into a prospective study (*n* = 532) and their association with all-cause mortality.

Parameter	Value	Association with all-cause mortality^a,b,c^
*Demographic data*		
Male gender, *n*, % of all	297 (56.0)	0.360
Age at the beginning of the study, years	61.2 (14.6–89.3)	<0.00001
RRT vintage prior to the study onset, years	2.2 (0.0–24.7)	0.309

*Cause of ESRD*		
Diabetic nephropathy, *n*, % of all	137 (25.8)	0.0002
Chronic glomerulonephritis, *n*, % of all	96 (18.0)	0.016
Hypertensive nephropathy, *n*, % of all	86 (16.2)	0.512
Chronic tubulointerstitial nephritis, *n*, % of all	67 (12.6)	0.981
Polycystic kidney disease, *n*, % of all	31 (5.8)	0.014
Kidney cancer, *n*, % of all	8 (1.5)	—
Urological diseases other than kidney cancer, *n*, % of all	28 (5.3)	—
Myeloma multiplex, *n*, % of all	6 (1.1)	—
Amyloidosis, *n*, % of all	7 (1.3)	—
Lupus nephritis, *n*, % of all	2 (0.4)	—
Autoimmune connective tissue disorders other than lupus nephritis, *n*, % of all	9 (1.7)	—
Systemic vasculitis, *n*, % of all	4 (0.8)	—
Rare, *n*, % of all	24 (4.5)	—
Unknown, *n*, % of all	27 (5.1)	—

*Clinical data*		
Coronary artery disease, *n*, % of all	212 (39.8)	<0.00001
Dyslipidemia, *n*, % of all	218 (41.0)	0.063
History of HBV infection (anti-HBc positivity), *n*, % of all	128 (24.1)	0.303
History of HCV infection (anti-HCV positivity), *n*, % of all	59 (11.1)	0.712
HBsAg positivity, *n*, % of all	15 (2.8)	0.397
HCV RNA positivity, *n*, % of all	36 (6.8)	0.664
Positive anti-HBs, *n*, % of all	457 (85.9)	0.013

*Type of RRT*		
LF-HD, *n*, % of all	277 (52.0)	0.853
HF-HD, *n*, % of all	217 (40.8)	0.390
HDF, *n*, % of all	38 (7.1)	0.201
HF-HD/HDF, *n*, % of all	255 (47.9)	0.853
PD as the first modality of RRT, *n*, % of all	17 (3.2)	0.165

*Laboratory data*		
ALT, IU/L	13.0 (2.0–131.0)	0.863
AST, IU/L	14.0 (3.0–177.0)	0.125
GGT, IU/L	26.0 (1.0–682)	0.089
ALP, U/L	97.0 (38.3–1684)	0.861
PTH, pg/mL	406 (12.7–3757)	0.0001
Ca, mg/dL	8.9 (5.4–11.7)	0.089
P, mg/dL	5.0 (2.2–11.3)	0.052

^a^The Kaplan-Meier analysis was conducted only for uniform parameters exceeding 10 individual reports.

^b^A multiple *P* value or a *P* value calculated using the log rank test, as appropriate.

^c^Associations were tested in relation to the tertiles of continuous variables.

ALP: alkaline phosphatase, ALT: alanine aminotransferase, Anti-HBc: antibody to core antigen of hepatitis B virus, Anti-HCV: antibody to hepatitis C virus, AST: aspartate aminotransferase, ESRD: end-stage renal disease, GGT: gamma-glutamyl transferase, HBsAg: surface antigen of hepatitis B virus, HBV: hepatitis B virus, HCV: hepatitis C virus, HDF: hemodiafiltration, HF-HD: high flux hemodialysis, LF-HD: low flux hemodialysis, PD: peritoneal dialysis, PTH: parathyroid hormone, RNA: ribonucleic acid, and RRT: renal replacement therapy.

Conversion factors to SI units are as follows: for alanine aminotransferase, 1 U/L = 0.0167 *µ*kat/L, for alkaline phosphatase, 1 U/L = 0.0167 *µ*kat/L, for aspartate aminotransferase, 1 U/L = 0.0167 *µ*kat/L, for calcium, 1 mg/dL = 0.25 mmol/L, for gamma-glutamyltransferase, 1 U/L = 0.0167 *µ*kat/L, for parathyroid hormone, 1 pg/mL = 1 ng/L, and for phosphorus, 1 mg/dL = 0.323 mmol/L.

**Table 2:** Characteristics of hemodialysis patients at the end of a 7-year prospective study.

Parameter	Value
*Demographic data*	
Total RRT vintage, years	7.4 (0.5–28.3)
RRT vintage on the prospective study, years	4.5 (0.1–7.0)
Movement to a noncollaborating dialysis center, *n*, % of all	7 (1.3)
Renal transplantation, *n*, % of all	66 (12.4)

*Causes of death* ^a^	
All, *n*, % of all	317 (59.6)
Cardiovascular, *n*, % of all	203 (38.2)
Cardiac, *n*, % of all	141 (26.5)
Sepsis/infection, *n*, % of all	39 (7.3)
Neoplasms, *n*, % of all	30 (5.6)
Rare/unknown, *n*, % of all	45 (8.5)

^a^Death rates are calculated in respect to all patients enrolled in the study (*n* = 532). Outcomes of 66 transplant recipients and 7 patients moving to other centers are not included.

RRT: renal replacement therapy.

**Table 3:** Statistical significance of differences in all-cause mortality evaluated by the Kaplan-Meier analysis for vitamin D signaling pathway genes and *CASR *in hemodialysis patients undergoing the 7-year prospective study.

Tested polymorphism	*N*	Major homozygotes versus heterozygotes versus minor homozygotes^a^	Dominant model of inheritance^b^	Recessive model of inheritance^b^	Additive model of inheritance^b^
*GC *rs7041	458	GG versus GT versus TT *P* = 0.692	TT + GT versus GG *P* = 0.822	TT versus GT + GG *P* = 0.320	TT versus GG *P* = 0.427
*GC *rs1155563	472	TT versus CT versus CC *P* = 0.342	CC + CT versus TT *P* = 0.420	CC versus CT + TT *P* = 0.275	CC versus TT *P* = 0.255
*GC *rs2298849	472	TT versus CT versus CC *P* = 0.136	CC + CT versus TT *P* = 0.024	CC versus CT + TT *P* = 0.829	CC versus TT *P* = 0.559
*RXRA *rs10881578	472	AA versus AG versus GG *P* = 0.252	GG + AG versus AA *P* = 0.151	GG versus AG + AA *P* = 0.587	GG versus AA *P* = 0.404
*RXRA *rs10776909	472	CC versus CT versus TT *P* = 0.848	TT + CT versus CC *P* = 0.672	TT versus CT + CC *P* = 0.908	TT versus CC *P* = 0.876
*RXRA *rs749759	464	GG versus AG versus AA *P* = 0.516	AA + AG versus GG *P* = 0.263	AA versus AG + GG *P* = 0.942	AA versus GG *P* = 0.830
*VDR *rs1544410	461	GG versus AG versus AA *P* = 0.555	AA + AG versus GG *P* = 0.372	AA versus AG + GG *P* = 0.471	AA versus GG *P* = 0.348
*VDR *rs2228570	449	CC versus CT versus TT *P* = 0.863	TT + CT versus CC *P* = 0.677	TT versus CT + CC *P* = 0.470	TT versus CC *P* = 0.493
*CASR *rs7652589	435	GG versus AG versus AA *P* = 0.255	AA + AG versus GG *P* = 0.200	AA versus AG + GG *P* = 0.352	AA versus GG *P* = 0.209

*CASR*: calcium-sensing receptor gene, *GC*: group-specific component gene, * RXRA*: retinoic X receptor alpha gene, and *VDR*: vitamin D receptor gene.

^a^Multiple-sample test *P*.

^b^Log rank test *P*.

**Table 4:** Statistical significance of differences in cardiovascular mortality evaluated by the Kaplan-Meier analysis for vitamin D signaling pathway genes and *CASR *in hemodialysis patients undergoing the 7-year prospective study.

Tested polymorphism	*N*	Major homozygotes versus heterozygotes versus minor homozygotes^a^	Dominant model of inheritance^b^	Recessive model of inheritance^b^	Additive model of inheritance^b^
*GC *rs7041	458	GG versus GT versus TT *P* = 0.329	TT + GT versus GG *P* = 0.475	TT versus GT + GG *P* = 0.648	TT versus GG *P* = 0.513
*GC *rs1155563	472	TT versus CT versus CC *P* = 0.316	CC + CT versus TT *P* = 0.542	CC versus CT + TT *P* = 0.992	CC versus TT *P* = 0.846
*GC *rs2298849	472	TT versus CT versus CC *P* = 0.906	CC + CT versus TT *P* = 0.797	CC versus CT + TT *P* = 0.921	CC versus TT *P* = 0.956
*RXRA *rs10881578	472	AA versus AG versus GG *P* = 0.778	GG + AG versus AA *P* = 0.617	GG versus AG + AA *P* = 0.391	GG versus AA *P* = 0.516
*RXRA *rs10776909	472	CC versus CT versus TT *P* = 0.294	TT + CT versus CC *P* = 0.176	TT versus CT + CC *P* = 0.595	TT versus CC *P* = 0.647
*RXRA *rs749759	464	GG versus AG versus AA *P* = 0.705	AA + AG versus GG *P* = 0.899	AA versus AG + GG *P* = 0.416	AA versus GG *P* = 0.429
*VDR *rs1544410	461	GG versus AG versus AA *P* = 0.760	AA + AG versus GG *P* = 0.098	AA versus AG + GG *P* = 0.976	AA versus GG *P* = 0.286
*VDR *rs2228570	449	CC versus CT versus TT *P* = 0.217	TT + CT versus CC *P* = 0.067	TT versus CT + CC *P* = 0.206	TT versus CC *P* = 0.043
*CASR *rs7652589	435	GG versus AG versus AA *P* = 0.119	AA + AG versus GG *P* = 0.781	AA versus AG + GG *P* = 0.090	AA versus GG *P* = 0.168

*CASR*: calcium-sensing receptor gene, *GC*: group-specific component gene, * RXRA*: retinoic X receptor alpha gene, and *VDR*: vitamin D receptor gene.

^a^Multiple-sample test *P*.

^b^Log rank test *P*.
