# Supplementary material for: Polymorphisms of Vitamin D Signaling Pathway Genes and Calcium-Sensing Receptor Gene in respect to Survival of Hemodialysis Patients: A Prospective Observational Study
Source: Int J Endocrinol. 2016 Aug 23;2016:2383216. doi: 10.1155/2016/2383216 (PMC5011523; doi:10.1155/2016/2383216)
Supplement: Supplementary file 1 — The Supplementary Material contains characteristics of the analyzed polymorphisms and conditions for their identification (Supplementary Tables 1-2), associations of vitamin D signaling pathway genes and CASR with infection-related and neoplasm-related mortalities (Supplementary Tables 3-4), characteristics of patients in respect to GC rs2298849 and VDR rs2228570 polymorphic variants (Supplementary Tables 5-6), haplotype frequencies in patients who died on HD and those who survived on HD for 7 years in respect to all-cause and cardiovascular mortality (Supplementary Tables 7-8), epistatic interactions between genes in patients who died on HD and those who survived on HD for 7 years in respect to all-cause and cardiovascular mortality (Supplementary Tables 9-10), and haplotype frequencies and epistatic interactionsbetweengenes in dyslipidemic HD patients and non-dyslipidemic HD subjects(Supplementary Table 11-12). Cumulative proportion surviving curves are presented for cardiovascular, infection-related and neoplasm-related mortalities in hemodialysis patients in respect to GC rs2298849 polymorphism (Supplementary Figures 1-3) as well as in respect to circulating 25(OH)D at the start of the prospective study (Supplementary Figure 4). [file 2383216.f1.docx]

**Polymorphisms of vitamin D signaling pathway genes and calcium sensing receptor gene in respect to survival of hemodialysis patients – a prospective observational study**

Alicja E. Grzegorzewska, Chair and Department of Nephrology, Transplantology and Internal Diseases, Poznan University of Medical Sciences, 60-355 Poznań, Przybyszewskiego 49, Poland; alicja_grzegorzewska@yahoo.com

Monika K. Świderska, Student Nephrology Research Group, Chair and Department of Nephrology, Transplantology and Internal Diseases, Poznan University of Medical Sciences, 60-355 Poznań, Przybyszewskiego 49, Poland; [monika.swi@gmail.com](mailto:monika.swi@gmail.com)

Adrianna Mostowska, Chair and Department of Biochemistry and Molecular Biology, Poznan University of Medical Sciences, 60-781 Poznań, Święcickiego 6, Poland; [amostowska@wp.pl](mailto:amostowska@wp.pl)

Wojciech Warchoł, Chair and Department of Biophysics, Poznan University of Medical Sciences, 60-780 Poznań, Grunwaldzka 6, Poland; [wwarchol@ump.edu.pl](mailto:wwarchol@ump.edu.pl)

Paweł P. Jagodziński, Chair and Department of Biochemistry and Molecular Biology, Poznan University of Medical Sciences, 60-781 Poznań, Święcickiego 6, Poland; [pjagodzi@ump.edu.pl](mailto:pjagodzi@ump.edu.pl)

The corresponding author:

Alicja E. Grzegorzewska, MD, PhD

Chair and Department of Nephrology, Transplantology and Internal Diseases,

Poznan University of Medical Sciences

60-355 Poznań, Przybyszewskiego 49, Poland

Fax: 48 61 8691 688, Phone: 48 618691700

E-mail: alicja_grzegorzewska@yahoo.com

*Supplementary Material*

*Supplementary Table 1*. Characteristics of the analyzed polymorphisms

| Gene symbol | rs no. | Location^a^ | Alleles^b^ | SNP function^c^ | MAF^d^ |
| --- | --- | --- | --- | --- | --- |
| *GC* | rs7041 | chr4:72618334 | G / T | Missense (p.Asp432Glu) | 0.417 |
| *GC* | rs1155563 | chr4:72643488 | C / T | Intron | 0.244 |
| *GC* | rs2298849 | chr4:72648851 | C / T | Intron | 0.199 |
| *RXRA* | rs10881578 | chr9:137232535 | A / G | Intron | 0.289 |
| *RXRA* | rs10776909 | chr9:137288746 | C / T | Intron | 0.215 |
| *RXRA* | rs749759 | chr9:137324652 | A / G | Intron | 0.234 |
| *VDR* | rs1544410 | chr12:48239835 | A / G | Intron | 0.404 |
| *VDR* | rs2228570 | chr12:48272895 | C / T | Missense (p.Met51Thr) | 0.378 |
| *CASR* | rs7652589 | chr3:121889088 | A / G | N/A | 0.359 |

^a^  - NCBI build 37 / hg19

^b^ - Underline denotes the minor allele

^c^ - According to the Single Nucleotide Polymorphism database (dbSNP)

^d^ - MAF, minor allele frequency calculated from 1000 Genomes project for EUR samples

Abbreviations: *GC* - group-specific component gene, MAF, minor allele frequency calculated from 1000 Genomes project for EUR samples, *RXRA* – retinoid X receptor alpha gene, SNP – single nucleotide polymorphism, UTR – untranslated region, *VDR* - vitamin D receptor gene

*Supplementary Table 2*. HRM and RFLP conditions for the identification of genotyped polymorphisms

|  |  |  |  |  |  | **HRM analysis** | **RFLP analysis** | |
| --- | --- | --- | --- | --- | --- | --- | --- | --- |
| **Gene symbol** | **rs no.** | **Alleles** | **Primers for PCR amplification  (5’ – 3’)** | **Annealing temp. (°C)** | **PCR product length (bp)** | **Melting temp. range (°C)** | **Restriction enzyme** | **Restriction fragment length (bp)** |
| *GC* | rs7041 | G/T | F: GGAGGTGAGTTTATGGAACAGC | 66.3 | 493 |  | HaeIII | G = 414 + 79 |
|  |  |  | R: GGCATTAAGCTGGTATGAGGTC |  |  |  |  | T = 493 |
|  | rs1155563 | C/T | F: GGTTATTCTAAGACTGTGCTCTTGC | 63.0 | 116 | 71 - 78 |  |  |
|  |  |  | R: ATGTGTTCTCACTGTTCGACTCC |  |  |  |  |  |
|  | rs2298849 | C/T | F: TCCACTGGCAAAACACATTAC | 60.6 | 118 | 73 - 83 |  |  |
|  |  |  | R: GGGACATCTGCATTTATCCTG |  |  |  |  |  |
| *RXRA* | rs10881578 | A/G | F: TCTTGAGCAATGCCAGCAG | 60.6 | 75 | 80 - 90 |  |  |
|  |  |  | R: CCACAGCTCACACATCCAATC |  |  |  |  |  |
|  | rs10776909 | C/T | F: CAGCCTGTGGCCTGCTCA | 60.6 | 95 | 82 - 92 |  |  |
|  |  |  | R: AACCTCCGGCCCTTGGAG |  |  |  |  |  |
|  | rs749759 | A/G | F: ATAGGGCTTGCCTGCCTAGA | 62.6 | 382 |  | BstXI | A = 382 |
|  |  |  | R: CTCCACCATAGCCCAAGTGA |  |  |  |  | G = 243 + 139 |
| *VDR* | rs1544410 | A/G | F: GGAGACACAGATAAGGAAATAC | 60.6 | 248 |  | FspI | A (B) = 248 |
|  |  |  | R: CCGCAAGAAACCTCAAATAACA |  |  |  |  | G (b) = 175+73 |
|  | rs2228570 | C/T | F: GCACTGACTCTGGCTCTGAC | 72.5 | 341 |  | FokI | C (F) = 341 |
|  |  |  | R: ACCCTCCTGCTCCTGTGGCT |  |  |  |  | T (f) = 282 + 59 |
| *CASR* | rs7652589 | G/A | F: ACTGCCCTCATCATTCCTTC | 61.0 | 178 | 77 - 92 |  |  |
|  |  |  | R: ATCATCCTCCCTGCAAGAAC |  |  |  |  |  |

Abbreviations: *CASR* - calcium-sensing receptor gene, *GC* - group-specific component gene, HRM analysis - High Resolution Melt analysis; RFLP analysis - Restriction Fragment Length Polymorphism analysis, *RXRA* - retinoic X receptor alpha gene, *VDR* - vitamin D receptor gene

*Supplementary Table* 3. Statistical significance of differences in infection-related mortality evaluated by the Kaplan-Meier analysis for vitamin D signaling pathway genes and *CASR* in hemodialysis patients undergoing the 7-year prospective study

| Tested polymorphism | N | Major homozytes vs. heterozygotes vs. minor homozygotes^a^ | Dominant model of inheritance^b^ | Recessive model of inheritance^b^ | Additive model of inheritance^b^ |
| --- | --- | --- | --- | --- | --- |
| *GC* rs7041 | 458 | GG vs. GT vs. TT  P = 0.899 | TT + GT vs. GG  P = 0.416 | TT vs. GT + GG  P = 0.435 | TT vs. GG  P = 0.358 |
| *GC* rs1155563 | 472 | TT vs. CT vs. CC  P = 0.947 | CC + CT vs. TT  P = 0.872 | CC vs. CT + TT  P = 0.557 | CC vs. TT  P = 0.502 |
| *GC* rs2298849 | 472 | TT vs. CT vs. CC  P = 0.122 | CC + CT vs. TT  P = 0.061 | CC vs. CT + TT  P = 0.199 | CC vs. TT  P = 0.165 |
| *RXRA* rs10881578 | 472 | AA vs. AG vs. GG  P = 0.488 | GG + AG vs. AA  P = 0.227 | GG vs. AG + AA  P = 0.535 | GG vs. AA  P = 0.454 |
| *RXRA* rs10776909 | 472 | CC vs. CT vs. TT  P = 0.045^c^ | TT + CT vs. CC  P = 0.089 | TT vs. CT + CC  P = 0.107 | TT vs. CC  P = 0.061 |
| *RXRA* rs749759 | 464 | GG vs. AG vs. AA  P = 0.574 | AA + AG vs. GG P = 0.599 | AA vs. AG + GG  P = 0.275 | AA vs. GG  P = 0.309 |
| *VDR* rs1544410 | 461 | GG vs. AG vs. AA P = 0.572 | AA + AG vs. GG P = 0.499 | AA vs. AG + GG  P = 0.461 | AA vs. GG  P = 0.798 |
| *VDR* rs2228570 | 449 | CC vs. CT vs. TT  P = 0.587 | TT + CT vs. CC  P = 0.747 | TT vs. CT + CC  P = 0.993 | TT vs. CC  P = 0.644 |
| *CASR* rs7652589 | 435 | GG vs. AG vs. AA P = 0.322 | AA + AG vs. GG P = 0.066 | AA vs. AG + GG  P = 0.561 | AA vs. GG  P = 0.383 |

Abbreviations: *CASR* – calcium-sensing receptor gene, *GC* – group-specific component gene,  *RXRA* – retinoic X receptor alpha gene, *VDR* – vitamin D receptor gene

a - Multiple-sample test P

b – Log rank test P

c – significance not confirmed in post hoc analyses

*Supplementary Table 4*. Statistical significance of differences in neoplasm-related mortality evaluated by the Kaplan-Meier analysis for vitamin D signaling pathway genes and *CASR* in hemodialysis patients undergoing the 7-year prospective study

| Tested polymorphism | N | Major homozytes vs. heterozygotes vs. minor homozygotes^a^ | Dominant model of inheritance^b^ | Recessive model of inheritance^b^ | Additive model of inheritance^b^ |
| --- | --- | --- | --- | --- | --- |
| *GC* rs7041 | 458 | GG vs. GT vs. TT  P = 0.980 | TT + GT vs. GG  P = 0.946 | TT vs. GT + GG  P = 0.889 | TT vs. GG  P = 0.959 |
| *GC* rs1155563 | 472 | TT vs. CT vs. CC  P = 0.856 | CC + CT vs. TT  P = 0.657 | CC vs. CT + TT  P = 0.576 | CC vs. TT  P = 0.850 |
| *GC* rs2298849 | 472 | TT vs. CT vs. CC  NA | CC + CT vs. TT  P = 0.310 | CC vs. CT + TT  NA | CC vs. TT  NA |
| *RXRA* rs10881578 | 472 | AA vs. AG vs. GG  P = 0.954 | GG + AG vs. AA  P = 0.660 | GG vs. AG + AA  P = 0.858 | GG vs. AA  P = 0.811 |
| *RXRA* rs10776909 | 472 | CC vs. CT vs. TT  P = 0.559 | TT + CT vs. CC  P = 0.926 | TT vs. CT + CC  P = 0.777 | TT vs. CC  P = 0.738 |
| *RXRA* rs749759 | 464 | GG vs. AG vs. AA  P = 0.573 | AA + AG vs. GG P = 0.409 | AA vs. AG + GG  P = 0.131 | AA vs. GG  P = 0.142 |
| *VDR* rs1544410 | 461 | GG vs. AG vs. AA P = 0.736 | AA + AG vs. GG P = 0.427 | AA vs. AG + GG  P = 0.968 | AA vs. GG  P = 0.750 |
| *VDR* rs2228570 | 449 | CC vs. CT vs. TT  P = 0.153 | TT + CT vs. CC  P = 0.312 | TT vs. CT + CC  P = 0.969 | TT vs. CC  P = 0.435 |
| *CASR* rs7652589 | 435 | GG vs. AG vs. AA P = 0.409 | AA + AG vs. GG P = 0.284 | AA vs. AG + GG  P = 0.602 | AA vs. GG  P = 0.352 |

Abbreviations: *CASR* – calcium-sensing receptor gene, *GC* – group-specific component gene, NA – not applicable,  *RXRA* – retinoic X receptor alpha gene, *VDR* – vitamin D receptor gene

a - Multiple-sample test P

b – Log rank test P

*Supplementary Table 5*. Characteristics of major allele homozygotes and patients bearing the minor allele in *GC* rs2298849

| Parameter | *GC* rs2298849 TT  N = 306 | *GC* rs2298849 CT + CC  N = 166 | P value^a^ |
| --- | --- | --- | --- |
| **Demographic data** |  |  |  |
| Male gender, n, % of all | 168 (54.9) | 96 (57.8) | 0.607 |
| Age at the beginning of the study, years | 61.1 (14.6 – 86.7) | 61.9 (17.5 – 89.3) | 0.580 |
| Age at the beginning of RRT, years | 58.8 (12.8 – 86.6) | 58.3 (11.1 – 86.5) | 0.939 |
| RRT vintage prior to the study onset, years | 2.0 (0.0 – 22.2) | 2.6 (0.0 – 24.7) | 0.023 |
| Total RRT vintage | 7.3 (0.5 – 28.3) | 7.4 (0.5 – 24.8) | 0.809 |
| RRT vintage on the prospective study | 4.8 (0.1 – 7.0) | 4.1 (0.2 – 7.0) | 0.009 |
| Time since RRT onset to renal transplantation | 4.3 (0.6 – 19.9) | 4.3 (0.7 – 14.3) | 0.801 |
| **Cause of ESRD** |  |  |  |
| Diabetic nephropathy, n, % of all | 74 (24.2) | 52 (31.3) | 0.117 |
| Chronic glomerulonephritis, n, % of all | 54 (17.6) | 23 (13.9) | 0.350 |
| Hypertensive nephropathy, n, % of all | 51 (16.7) | 30 (18.1) | 0.796 |
| Chronic tubulointerstitial nephritis, n, % of all | 38 (12.4) | 15 (9.0) | 0.338 |
| Polycystic kidney disease, n, % of all | 19 (6.2) | 10 (6.0) | 0.904 |
| **Clinical data** |  |  |  |
| Coronary artery disease, n, % of all | 122 (39.9) | 70 (42.2) | 0.698 |
| Myocardial infarction, n, % of all | 69 (22.5) | 41 (24.7) | 0.679 |
| Responders to HBV vaccination among HBV non-infected patients | 203/233 (87.1) | 106/122 (86.9) | 0.918 |
| Maximum anti-HBs titer among vaccine responders | 335 (10 – 4262) | 410 (10.4 – 5342) | 0.601 |
| History of HBV infection (anti-HBc positivity), n, % of all | 73 (23.9) | 44 (26.5) | 0.600 |
| History of HCV infection (anti-HCV positivity), n, % of all | 31 (10.1) | 23 (13.9) | 0.288 |
| HBsAg positivity, n, % of all | 8 (2.6) | 6 (3.6) | 0.743 |
| HCV RNA positivity, n, % of all | 20 (6.5) | 14 (8.4) | 0.565 |
| Renal transplantation, n, % of all | 32 (10.5) | 28 (16.9) | 0.064 |
| Cinacalcet treatment/ Parathyroidectomy, n, % of all | 78 (25.5) | 48 (28.9) | 0.488 |
| Dyslipidemia, n, % of all | 120 (39.2) | 73 (44.0) | 0.365 |
| **Type of RRT** |  |  |  |
| LF-HD, n, % of all | 163 (53.3) | 88 (53.0) | 0.965 |
| HF-HD, n, % of all | 120 (39.2) | 64 (38.6) | 0.967 |
| HDF, n, % of all | 23 (7.5) | 14 (8.4) | 0.861 |
| HF-HD/HDF, n, % of all | 143 (46.7) | 78 (47.0) | 0.965 |
| PD as the first modality of RRT, n, % of all | 9 (2.9) | 7 (4.2) | 0.642 |
| **Laboratory data** |  |  |  |
| ALT, IU/L | 13 (3 – 131) | 13 (2 – 49) | 0.438 |
| AST, IU/L | 14 (3 – 97) | 15 (4 – 177) | 0.564 |
| GGT, IU/L | 26 (1 – 513) | 26.5 (1 – 682) | 0.181 |
| ALP, U/L | 97 (42.5 – 1684) | 97.6 (38.3 – 680.5) | 0.732 |
| PTH, pg/mL | 426 (12.7 – 3757) | 448.5 (48.3 – 2607.3) | 0.388 |
| Ca, mg/dL | 8.9 (5.4 – 11.7) | 8.9 (6.8 – 11.2) | 0.651 |
| P, mg/dL | 5.1 (2.2 – 11.3) | 5.0 (2.2 – 10.5) | 0.726 |
| **Causes of death** |  |  |  |
| All, n, % of all | 179 (58.5) | 107 (64.5) | 0.243 |
| Cardiovascular, n, % of all | 115 (37.6) | 68 (41.0) | 0.535 |
| Cardiac, n, % of all | 78 (25.5) | 45 (27.1) | 0.785 |
| Sepsis/infection, n, % of all | 21 (6.9) | 14 (8.4) | 0.661 |
| Neoplasms, n, % of all | 17 (5.6) | 9 (5.4) | 0.881 |
| Rare/unknown, n, % of all | 26 (8.5) | 16 (9.6) | 0.805 |

a – Mann-Whitney U test was used for comparison of continuous variables. Chi square test with Yates correction was applied for comparison of dichotomous variables.

Abbreviations:

Abbreviations: ALP - alkaline phosphatase, ALT – alanine aminotransferase, Anti-HBc - antibodies to core antigen of hepatitis B virus, Anti-HBs - antibodies to surface antigen of hepatitis B virus, AST - aspartate aminotransferase, ESRD – end-stage renal disease, GGT - gamma-glutamyl transferase, HBsAg - surface antigen of hepatitis B virus, HBV - hepatitis B virus, HCV - hepatitis C virus, HDF - hemodiafiltration , HF-HD - high flux hemodialysis, LF-HD - low flux hemodialysis, N - number of patients, PD - peritoneal dialysis, PTH - parathyroid hormone, RNA - ribonucleic acid, RRT - renal replacement therapy

*Supplementary Table 6*. Characteristics of major allele homozygotes and minor allele homozygotes in *VDR* rs2228570

| Parameter | *VDR* rs2228570 CC  N = 128 | *VDR* rs2228570 TT  N = 94 | P value^a^ |
| --- | --- | --- | --- |
| **Demographic data** |  |  |  |
| Male gender, n, % of all | 69 (53.9) | 50 (53.2) | 0.976 |
| Age at the beginning of the study, years | 60.9 (20.4 – 86.9) | 63.3 (17.5 – 85.6) | 0.206 |
| Age at the beginning of RRT, years | 57.7 (11.1 – 85.2) | 60.7 (16.4 – 83.0) | 0.298 |
| RRT vintage prior to the study onset, years | 1.9 (0.0 – 22.2) | 2.3 (0.0 – 14.3) | 0.545 |
| Total RRT vintage | 7.2 (0.5 – 28.3) | 7.5 (0.5 – 18.1) | 0.651 |
| RRT vintage on the prospective study | 4.2 (0.3 – 7.0) | 4.5 (0.3 – 7.0) | 0.889 |
| Time since RRT onset to renal transplantation | 4.3 (0.6 -12.0) | 3.8 (0.7 – 14.3) | 0.580 |
| **Cause of ESRD** |  |  |  |
| Diabetic nephropathy, n, % of all | 30 (23.4) | 24 (25.5) | 0.841 |
| Chronic glomerulonephritis, n, % of all | 15 (11.7) | 13 (13.8) | 0.792 |
| Hypertensive nephropathy, n, % of all | 20 (15.6) | 17 (18.1) | 0.761 |
| Chronic tubulointerstitial nephritis, n, % of all | 14 (10.9) | 12 (12.8) | 0.836 |
| Polycystic kidney disease, n, % of all | 11 (8.6) | 4 (4.3) | 0.316 |
| **Clinical data** |  |  |  |
| Coronary artery disease, n, % of all | 49 (38.3) | 35 (37.2) | 0.985 |
| Myocardial infarction, n, % of all | 30 (23.4) | 22 (23.4) | 0.877 |
| Responders to HBV vaccination among HBV non-infected patients | 83/100 (0.83) | 56/67 (0.84) | 0.910 |
| Maximum anti-HBs titer among vaccine responders | 291.8 (14 – 5342) | 443.8 (10.5 – 4262) | 0.392 |
| History of HBV infection (anti-HBc positivity), n, % of all | 28 (21.9) | 27 (28.7) | 0.312 |
| History of HCV infection (anti-HCV positivity), n, % of all | 15 (11.7) | 10 (10.6) | 0.971 |
| HBsAg positivity, n, % of all | 5 (3.9) | 4 (4.3) | 0.831 |
| HCV RNA positivity, n, % of all | 7 (5.5) | 6 (6.4) | 0.998 |
| Renal transplantation, n, % of all | 17 (13.3) | 12 (12.8) | 0.929 |
| Cinacalcet treatment, n, % of all | 27 (21.1) | 22 (23.4) | 0.805 |
| Parathyroidectomy, n, % of all | 7 (5.5) | 4 (4.3) | 0.921 |
| Cinacalcet treatment/ Parathyroidectomy, n, % of all | 34 (26.6) | 26 (27.7) | 0.977 |
| Dyslipidemia, n, % of all | 59 (46.1) | 30 (31.9) | 0.047 |
| **Type of RRT** |  |  |  |
| LF-HD, n, % of all | 66 (51.6) | 56 (59.6) | 0.294 |
| HF-HD, n, % of all | 50 (39.1) | 32 (34.0) | 0.532 |
| HDF, n, % of all | 12 (9.4) | 6 (6.4) | 0.577 |
| HF-HD/HDF, n, % of all | 62 (48.4) | 38 (40.4) | 0.294 |
| PD as the first modality of RRT, n, % of all | 3 (2.3) | 1 (1.1) | 0.843 |
| **Laboratory data** |  |  |  |
| ALT, IU/L | 13 (4 – 126) | 13.5 (2 – 131) | 0.518 |
| AST, IU/L | 14 (5 – 97) | 15 (4 – 97) | 0.124 |
| GGT, IU/L | 26 (4 – 513) | 26 (1 – 308) | 0.840 |
| ALP, U/L | 87.5 (42.5 – 680.5) | 94.5 (42.5 – 1353.3) | 0.642 |
| PTH, pg/mL | 430.4 (21.5 – 3757) | 481.8 (16.8 – 2735.5) | 0.405 |
| Ca, mg/dL | 9.0 ± 0.8 | 9.0 ± 0.7 | 0.885^b^ |
| P, mg/dL | 5.1 (2.2 – 10.5) | 5.0 (2.5 – 11.2) | 0.776 |
| **Causes of death** |  |  |  |
| All, n, % of all | 72 (56.3) | 60 (63.8) | 0.318 |
| Cardiovascular, n, % of all | 48 (37.5) | 38 (40.4) | 0.762 |
| Cardiac, n, % of all | 34 (26.6) | 25 (26.6) | 0.882 |
| Sepsis/infection, n, % of all | 6 (4.7) | 7 (7.4) | 0.565 |
| Neoplasms, n, % of all | 7 (5.5) | 5 (5.3) | 0.801 |
| Rare/unknown, n, % of all | 11 (8.6) | 10 (10.6) | 0.778 |

a – Mann-Whitney U test was used for comparison of continuous variables. Chi square test with Yates correction was applied for comparison of dichotomous variables.

b – Student’s T test

Abbreviations:

Abbreviations: ALP - alkaline phosphatase, ALT – alanine aminotransferase, Anti-HBc - antibodies to core antigen of hepatitis B virus, Anti-HBs - antibodies to surface antigen of hepatitis B virus, AST - aspartate aminotransferase, ESRD – end-stage renal disease, GGT - gamma-glutamyl transferase, HBsAg - surface antigen of hepatitis B virus, HBV - hepatitis B virus, HCV - hepatitis C virus, HDF - hemodiafiltration , HF-HD - high flux hemodialysis, LF-HD - low flux hemodialysis, N - number of patients, PD - peritoneal dialysis, PTH - parathyroid hormone, RNA - ribonucleic acid, RRT - renal replacement therapy

*Supplementary Table 7*. Haplotype frequencies in patients who died on HD (cases) and those who survived on HD for 7 years (controls)

| **Polymorphisms** | **Haplotype** | **Freq.** | **Case,Control Frequencies** | **Chi Square** | **P Value** | **P_corr_ Value^1^** |
| --- | --- | --- | --- | --- | --- | --- |
| ***VDR*** |  |  |  |  |  |  |
| rs1544410_rs2228570 | GC | 0.308 | 0.299, 0.330 | 0.718 | 0.397 | 0.733 |
|  | GT | 0.289 | 0.307, 0.246 | 3.036 | 0.081 | 0.157 |
|  | AC | 0.228 | 0.223, 0.238 | 0.203 | 0.652 | 0.958 |
|  | AT | 0.175 | 0.170, 0.186 | 0.303 | 0.582 | 0.913 |
| ***RXRA*** |  |  |  |  |  |  |
| rs10881578_rs10776909 | AC | 0.619 | 0.627, 0.600 | 0.528 | 0.467 | 0.835 |
|  | GC | 0.168 | 0.166, 0.172 | 0.045 | 0.832 | 0.997 |
|  | GT | 0.108 | 0.101, 0.125 | 1.011 | 0.315 | 0.666 |
|  | AT | 0.104 | 0.105, 0.102 | 0.016 | 0.899 | 1.000 |
| rs10776909_rs749759 | CG | 0.658 | 0.666, 0.639 | 0.56 | 0.454 | 0.850 |
|  | TA | 0.141 | 0.133, 0.159 | 0.983 | 0.322 | 0.706 |
|  | CA | 0.130 | 0.128, 0.134 | 0.059 | 0.808 | 0.994 |
|  | TG | 0.072 | 0.074, 0.068 | 0.076 | 0.783 | 0.990 |
| rs10881578_rs10776909_rs749759 | ACG | 0.507 | 0.517, 0.482 | 0.806 | 0.369 | 0.93 |
|  | GCG | 0.151 | 0.149, 0.157 | 0.083 | 0.773 | 1.000 |
|  | ACA | 0.113 | 0.111, 0.119 | 0.108 | 0.743 | 1.000 |
|  | ATA | 0.071 | 0.072, 0.069 | 0.019 | 0.891 | 1.000 |
|  | GTA | 0.070 | 0.061, 0.091 | 2.335 | 0.127 | 0.495 |
|  | GTG | 0.039 | 0.040, 0.035 | 0.129 | 0.720 | 1.000 |
|  | ATG | 0.033 | 0.033, 0.033 | 0.004 | 0.949 | 1.000 |
|  | GCA | 0.017 | 0.017, 0.015 | 0.062 | 0.804 | 1.000 |
| ***VDB*** |  |  |  |  |  |  |
| rs7041_rs1155563 | GT | 0.497 | 0.499, 0.494 | 0.013 | 0.908 | 0.998 |
|  | TC | 0.279 | 0.275, 0.290 | 0.187 | 0.665 | 0.969 |
|  | TT | 0.160 | 0.154, 0.175 | 0.568 | 0.451 | 0.862 |
|  | GC | 0.063 | 0.073, 0.041 | 2.883 | 0.090 | 0.927 |
| rs1155563_rs2298849 | TT | 0.492 | 0.475, 0.531 | 2.109 | 0.147 | 0.417 |
|  | CT | 0.309 | 0.306, 0.316 | 0.091 | 0.763 | 0.994 |
|  | TC | 0.166 | 0.177, 0.139 | 1.859 | 0.173 | 0.474 |
|  | CC | 0.034 | 0.042, 0.014 | 3.954 | 0.047 | 0.165 |
| rs7041_rs1155563_rs2298849 | GTT | 0.362 | 0.354, 0.380 | 0.494 | 0.482 | 0.989 |
|  | TCT | 0.258 | 0.249, 0.277 | 0.701 | 0.403 | 0.972 |
|  | GTC | 0.135 | 0.145, 0.114 | 1.357 | 0.244 | 0.829 |
|  | TTT | 0.130 | 0.121, 0.151 | 1.414 | 0.234 | 0.813 |
|  | GCT | 0.051 | 0.057, 0.038 | 1.169 | 0.280 | 0.876 |
|  | TTC | 0.031 | 0.033, 0.024 | 0.486 | 0.486 | 0.991 |
|  | TCC | 0.021 | 0.025, 0.012 | 1.427 | 0.232 | 0.808 |
|  | GCC | 0.012 | 0.016, 0.003 | 2.521 | 0.112 | 0.503 |
|  |  |  |  |  |  |  |
| ^1^p value calculated using permutation test and a total of 1,000 permutations | | | |  |  |  |

*Supplementary Table 8*. Haplotype frequencies in patients who died on HD due to cardiovascular diseases (cases) and those who survived on HD for 7 years (controls)

| **Polymorphisms** | **Haplotype** | **Freq.** | **Case,Control Frequencies** | **Chi Square** | **P Value** | **P_corr_ Value^1^** |
| --- | --- | --- | --- | --- | --- | --- |
| ***VDR*** |  |  |  |  |  |  |
| rs1544410_rs2228570 | GC | 0.322 | 0.311, 0.339 | 0.517 | 0.472 | 0.792 |
|  | GT | 0.261 | 0.277, 0.237 | 1.213 | 0.271 | 0.535 |
|  | AC | 0.222 | 0.218, 0.228 | 0.086 | 0.769 | 0.975 |
|  | AT | 0.195 | 0.194, 0.196 | 0.004 | 0.949 | 1.000 |
| ***RXRA*** |  |  |  |  |  |  |
| rs10881578_rs10776909 | AC | 0.618 | 0.630, 0.601 | 0.541 | 0.462 | 0.839 |
|  | GC | 0.182 | 0.188, 0.172 | 0.24 | 0.624 | 0.956 |
|  | GT | 0.107 | 0.095, 0.125 | 1.426 | 0.233 | 0.540 |
|  | AT | 0.093 | 0.087, 0.102 | 0.372 | 0.542 | 0.904 |
| rs10776909_rs749759 | CG | 0.663 | 0.680, 0.637 | 1.181 | 0.277 | 0.637 |
|  | CA | 0.137 | 0.138, 0.135 | 0.009 | 0.924 | 1.000 |
|  | TA | 0.129 | 0.110, 0.158 | 3.034 | 0.082 | 0.220 |
|  | TG | 0.071 | 0.072, 0.069 | 0.021 | 0.884 | 0.996 |
| rs10881578_rs10776909_rs749759 | ACG | 0.505 | 0.520, 0.483 | 0.777 | 0.378 | 0.948 |
|  | GCG | 0.157 | 0.159, 0.154 | 0.032 | 0.858 | 1.000 |
|  | ACA | 0.113 | 0.111, 0.118 | 0.076 | 0.783 | 1.000 |
|  | GTA | 0.070 | 0.056, 0.091 | 2.813 | 0.094 | 0.344 |
|  | ATA | 0.059 | 0.053, 0.067 | 0.473 | 0.491 | 0.987 |
|  | GTG | 0.037 | 0.039, 0.035 | 0.084 | 0.772 | 1.000 |
|  | ATG | 0.034 | 0.034, 0.034 | 0.004 | 0.949 | 1.000 |
|  | GCA | 0.024 | 0.028, 0.017 | 0.7 | 0.403 | 0.963 |
| ***VDB*** |  |  |  |  |  |  |
| rs7041_rs1155563 | GT | 0.494 | 0.493, 0.494 | 0.0 | 0.983 | 1.000 |
|  | TC | 0.280 | 0.273, 0.290 | 0.2 | 0.654 | 0.973 |
|  | TT | 0.166 | 0.161, 0.175 | 0.226 | 0.635 | 0.964 |
|  | GC | 0.060 | 0.073, 0.041 | 2.668 | 0.102 | 0.317 |
| rs1155563_rs2298849 | TT | 0.500 | 0.479, 0.531 | 1.545 | 0.214 | 0.579 |
|  | CT | 0.307 | 0.301, 0.316 | 0.161 | 0.688 | 0.967 |
|  | TC | 0.160 | 0.174, 0.138 | 1.399 | 0.237 | 0.607 |
|  | CC | 0.033 | 0.045, 0.015 | 4.344 | 0.037 | 0.141 |
| rs7041_rs1155563_rs2298849 | GTT | 0.360 | 0.347, 0.378 | 0.612 | 0.434 | 0.981 |
|  | TCT | 0.259 | 0.247, 0.277 | 0.695 | 0.404 | 0.972 |
|  | TTT | 0.140 | 0.131, 0.154 | 0.601 | 0.438 | 0.984 |
|  | GTC | 0.134 | 0.146, 0.115 | 1.201 | 0.273 | 0.891 |
|  | GCT | 0.049 | 0.055, 0.038 | 0.895 | 0.344 | 0.946 |
|  | TTC | 0.026 | 0.029, 0.022 | 0.271 | 0.603 | 0.998 |
|  | TCC | 0.021 | 0.026, 0.012 | 1.346 | 0.246 | 0.846 |
|  | GCC | 0.012 | 0.018, 0.003 | 2.872 | 0.090 | 0.463 |
|  |  |  |  |  |  |  |
| ^1^p value calculated using permutation test and a total of 1,000 permutations | | | |  |  |  |

*Supplementary Table 9*. Epistatic interactions between genes in patients who died on HD and those who survived on HD for 7 years

|  |  |  |  |  |  |  | **FDR-adjusted** |
| --- | --- | --- | --- | --- | --- | --- | --- |
| **CHR1** | **SNP1** | **CHR2** | **SNP2** | **OR_INT** | **STAT** | **P-value** | **p-values** |
| 3 | rs7652589 | 4 | rs7041 | 1.109 | 0.200 | 0.655 | 0.882 |
| 3 | rs7652589 | 4 | rs1155563 | 0.829 | 0.701 | 0.403 | 0.764 |
| 3 | rs7652589 | 4 | rs2298849 | 1.108 | 0.123 | 0.726 | 0.882 |
| 3 | rs7652589 | 9 | rs10881578 | 0.911 | 0.134 | 0.715 | 0.882 |
| 3 | rs7652589 | 9 | rs10776909 | 1.209 | 0.503 | 0.478 | 0.819 |
| 3 | rs7652589 | 9 | rs749759 | 0.736 | 1.336 | 0.248 | 0.638 |
| 3 | rs7652589 | 12 | rs1544410 | 0.739 | 1.662 | 0.197 | 0.638 |
| 3 | rs7652589 | 12 | rs2228570 | 0.930 | 0.083 | 0.774 | 0.896 |
| 4 | rs7041 | 4 | rs1155563 | 0.693 | 2.703 | 0.100 | 0.638 |
| 4 | rs7041 | 4 | rs2298849 | 1.472 | 1.711 | 0.191 | 0.638 |
| 4 | rs7041 | 9 | rs10881578 | 0.646 | 3.015 | 0.082 | 0.638 |
| 4 | rs7041 | 9 | rs10776909 | 1.428 | 1.757 | 0.185 | 0.638 |
| 4 | rs7041 | 9 | rs749759 | 1.044 | 0.032 | 0.857 | 0.900 |
| 4 | rs7041 | 12 | rs1544410 | 0.900 | 0.233 | 0.630 | 0.882 |
| 4 | rs7041 | 12 | rs2228570 | 1.181 | 0.571 | 0.450 | 0.810 |
| 4 | rs1155563 | 4 | rs2298849 | 1.632 | 1.927 | 0.165 | 0.638 |
| 4 | rs1155563 | 9 | rs10881578 | 0.696 | 2.436 | 0.119 | 0.638 |
| 4 | rs1155563 | 9 | rs10776909 | 1.140 | 0.280 | 0.597 | 0.882 |
| 4 | rs1155563 | 9 | rs749759 | 0.972 | 0.016 | 0.900 | 0.900 |
| 4 | rs1155563 | 12 | rs1544410 | 0.869 | 0.421 | 0.517 | 0.846 |
| 4 | rs1155563 | 12 | rs2228570 | 0.690 | 3.039 | 0.081 | 0.638 |
| 4 | rs2298849 | 9 | rs10881578 | 0.947 | 0.032 | 0.858 | 0.900 |
| 4 | rs2298849 | 9 | rs10776909 | 1.088 | 0.067 | 0.796 | 0.896 |
| 4 | rs2298849 | 9 | rs749759 | 0.675 | 1.871 | 0.171 | 0.638 |
| 4 | rs2298849 | 12 | rs1544410 | 0.891 | 0.158 | 0.691 | 0.882 |
| 4 | rs2298849 | 12 | rs2228570 | 0.901 | 0.132 | 0.717 | 0.882 |
| 9 | rs10881578 | 9 | rs10776909 | 0.697 | 1.544 | 0.214 | 0.638 |
| 9 | rs10881578 | 9 | rs749759 | 0.788 | 0.841 | 0.359 | 0.760 |
| 9 | rs10881578 | 12 | rs1544410 | 0.741 | 1.413 | 0.235 | 0.638 |
| 9 | rs10881578 | 12 | rs2228570 | 0.967 | 0.019 | 0.891 | 0.900 |
| 9 | rs10776909 | 9 | rs749759 | 0.916 | 0.114 | 0.735 | 0.882 |
| 9 | rs10776909 | 12 | rs1544410 | 0.766 | 0.910 | 0.340 | 0.760 |
| 9 | rs10776909 | 12 | rs2228570 | 0.776 | 0.889 | 0.346 | 0.760 |
| 9 | rs749759 | 12 | rs1544410 | 0.530 | 5.612 | 0.018 | 0.638 |
| 9 | rs749759 | 12 | rs2228570 | 0.806 | 0.759 | 0.384 | 0.764 |
| 12 | rs1544410 | 12 | rs2228570 | 0.669 | 2.802 | 0.094 | 0.638 |

| CHR1 Chromosome of first SNP | |
| --- | --- |
| SNP1 Identifier for first SNP |  |
| CHR2 Chromosome of second SNP | |
| SNP2 Identifier for second SNP | |
| OR_INT Odds ratio for interaction | |
| STAT Chi-square statistic, 1df |  |
| P Asymptotic p-value |  |

*Supplementary Table 10*. Epistatic interactions between genes in patients who died on HD due to cardiovascular diseases and those who survived on HD for 7 years

|  |  |  |  |  |  |  | **FDR-adjusted** |
| --- | --- | --- | --- | --- | --- | --- | --- |
| **CHR1** | **SNP1** | **CHR2** | **SNP2** | **OR_INT** | **STAT** | **P-value** | **p-values** |
| 3 | rs7652589 | 4 | rs7041 | 1.061 | 0.057 | 0.812 | 0.969 |
| 3 | rs7652589 | 4 | rs1155563 | 0.768 | 1.180 | 0.277 | 0.930 |
| 3 | rs7652589 | 4 | rs2298849 | 1.022 | 0.005 | 0.946 | 0.969 |
| 3 | rs7652589 | 9 | rs10881578 | 0.848 | 0.347 | 0.556 | 0.969 |
| 3 | rs7652589 | 9 | rs10776909 | 1.186 | 0.314 | 0.575 | 0.969 |
| 3 | rs7652589 | 9 | rs749759 | 0.875 | 0.206 | 0.650 | 0.969 |
| 3 | rs7652589 | 12 | rs1544410 | 0.756 | 1.152 | 0.283 | 0.930 |
| 3 | rs7652589 | 12 | rs2228570 | 0.951 | 0.032 | 0.857 | 0.969 |
| 4 | rs7041 | 4 | rs1155563 | 0.742 | 1.573 | 0.210 | 0.930 |
| 4 | rs7041 | 4 | rs2298849 | 1.385 | 1.087 | 0.297 | 0.930 |
| 4 | rs7041 | 9 | rs10881578 | 0.646 | 2.796 | 0.095 | 0.930 |
| 4 | rs7041 | 9 | rs10776909 | 1.197 | 0.372 | 0.542 | 0.969 |
| 4 | rs7041 | 9 | rs749759 | 0.916 | 0.117 | 0.733 | 0.969 |
| 4 | rs7041 | 12 | rs1544410 | 0.867 | 0.393 | 0.531 | 0.969 |
| 4 | rs7041 | 12 | rs2228570 | 1.231 | 0.770 | 0.380 | 0.930 |
| 4 | rs1155563 | 4 | rs2298849 | 1.675 | 2.026 | 0.155 | 0.930 |
| 4 | rs1155563 | 9 | rs10881578 | 0.694 | 2.274 | 0.132 | 0.930 |
| 4 | rs1155563 | 9 | rs10776909 | 0.963 | 0.018 | 0.893 | 0.969 |
| 4 | rs1155563 | 9 | rs749759 | 0.943 | 0.052 | 0.819 | 0.969 |
| 4 | rs1155563 | 12 | rs1544410 | 0.756 | 1.520 | 0.218 | 0.930 |
| 4 | rs1155563 | 12 | rs2228570 | 0.784 | 1.138 | 0.286 | 0.930 |
| 4 | rs2298849 | 9 | rs10881578 | 1.012 | 0.001 | 0.969 | 0.969 |
| 4 | rs2298849 | 9 | rs10776909 | 1.085 | 0.052 | 0.820 | 0.969 |
| 4 | rs2298849 | 9 | rs749759 | 0.804 | 0.510 | 0.475 | 0.969 |
| 4 | rs2298849 | 12 | rs1544410 | 0.970 | 0.010 | 0.921 | 0.969 |
| 4 | rs2298849 | 12 | rs2228570 | 0.875 | 0.189 | 0.664 | 0.969 |
| 9 | rs10881578 | 9 | rs10776909 | 0.705 | 1.254 | 0.263 | 0.930 |
| 9 | rs10881578 | 9 | rs749759 | 0.929 | 0.069 | 0.793 | 0.969 |
| 9 | rs10881578 | 12 | rs1544410 | 0.877 | 0.237 | 0.626 | 0.969 |
| 9 | rs10881578 | 12 | rs2228570 | 1.026 | 0.010 | 0.922 | 0.969 |
| 9 | rs10776909 | 9 | rs749759 | 0.742 | 0.924 | 0.337 | 0.930 |
| 9 | rs10776909 | 12 | rs1544410 | 0.907 | 0.101 | 0.751 | 0.969 |
| 9 | rs10776909 | 12 | rs2228570 | 0.814 | 0.475 | 0.491 | 0.969 |
| 9 | rs749759 | 12 | rs1544410 | 0.640 | 2.391 | 0.122 | 0.930 |
| 9 | rs749759 | 12 | rs2228570 | 0.779 | 0.849 | 0.357 | 0.930 |
| 12 | rs1544410 | 12 | rs2228570 | 0.802 | 0.748 | 0.387 | 0.930 |

| CHR1 Chromosome of first SNP |
| --- |
| SNP1 Identifier for first SNP |
| CHR2 Chromosome of second SNP |
| SNP2 Identifier for second SNP |
| OR_INT Odds ratio for interaction |
| STAT Chi-square statistic, 1df |
| P Asymptotic p-value |

*Supplementary Table 11*. Haplotype frequencies in dyslipidemic HD patients (cases) and non-dyslipidemic HD subjects (controls)

| **Polymorphisms** | **Haplotype** | **Freq.** | **Case,Control Frequencies** | **Chi Square** | **P Value** | **P_corr_ Value^1^** |
| --- | --- | --- | --- | --- | --- | --- |
| ***VDR*** |  |  |  |  |  |  |
| rs1544410_rs2228570 | GC | 0.322 | 0.316, 0.328 | 0.124 | 0.725 | 0.972 |
|  | GT | 0.289 | 0.279, 0.299 | 0.371 | 0.542 | 0.888 |
|  | **AC** | **0.220** | **0.261, 0.177** | **7.679** | **0.006** | **0.005** |
|  | AT | 0.169 | 0.144, 0.196 | 3.554 | 0.059 | 0.110 |
| ***RXRA*** |  |  |  |  |  |  |
| rs10881578_rs10776909 | AC | 0.628 | 0.616, 0.641 | 0.481 | 0.488 | 0.854 |
|  | GC | 0.162 | 0.159, 0.166 | 0.057 | 0.812 | 0.989 |
|  | GT | 0.113 | 0.109, 0.117 | 0.117 | 0.732 | 0.975 |
|  | AT | 0.097 | 0.115, 0.077 | 3.23 | 0.072 | 0.178 |
| rs10776909_rs749759 | CG | 0.669 | 0.646, 0.692 | 1.772 | 0.183 | 0.469 |
|  | TA | 0.145 | 0.169, 0.121 | 3.536 | 0.060 | 0.180 |
|  | CA | 0.122 | 0.129, 0.115 | 0.388 | 0.534 | 0.920 |
|  | TG | 0.064 | 0.056, 0.073 | 0.955 | 0.328 | 0.710 |
| rs10881578_rs10776909_rs749759 | ACG | 0.523 | 0.504, 0.542 | 1.094 | 0.296 | 0.858 |
|  | GCG | 0.146 | 0.142, 0.150 | 0.091 | 0.763 | 1.000 |
|  | ACA | 0.106 | 0.112, 0.100 | 0.315 | 0.575 | 0.997 |
|  | GTA | 0.073 | 0.077, 0.068 | 0.244 | 0.621 | 0.998 |
|  | ATA | 0.072 | 0.092, 0.052 | 4.325 | 0.038 | 0.122 |
|  | GTG | 0.040 | 0.031, 0.050 | 1.631 | 0.202 | 0.701 |
|  | ATG | 0.024 | 0.024, 0.024 | 0.002 | 0.964 | 1.000 |
|  | GCA | 0.016 | 0.017, 0.015 | 0.072 | 0.788 | 1.000 |
| ***GC*** |  |  |  |  |  |  |
| rs7041_rs1155563 | GT | 0.499 | 0.489, 0.509 | 0.313 | 0.576 | 0.935 |
|  | TC | 0.287 | 0.304, 0.270 | 1.023 | 0.312 | 0.690 |
|  | TT | 0.156 | 0.151, 0.161 | 0.121 | 0.729 | 0.985 |
|  | GC | 0.058 | 0.056, 0.060 | 0.05 | 0.824 | 0.996 |
| rs1155563_rs2298849 | TT | 0.498 | 0.475, 0.522 | 1.682 | 0.195 | 0.491 |
|  | CT | 0.308 | 0.310, 0.306 | 0.015 | 0.902 | 1.000 |
|  | TC | 0.156 | 0.165, 0.147 | 0.448 | 0.503 | 0.870 |
|  | CC | 0.038 | 0.050, 0.025 | 3.354 | 0.067 | 0.205 |
| rs7041_rs1155563_rs2298849 | GTT | 0.369 | 0.354, 0.385 | 0.745 | 0.388 | 0.945 |
|  | TCT | 0.263 | 0.267, 0.258 | 0.086 | 0.769 | 1.000 |
|  | GTC | 0.129 | 0.134, 0.124 | 0.155 | 0.694 | 1.000 |
|  | TTT | 0.128 | 0.120, 0.137 | 0.523 | 0.470 | 0.980 |
|  | GCT | 0.046 | 0.043, 0.048 | 0.09 | 0.764 | 1.000 |
|  | TTC | 0.028 | 0.032, 0.023 | 0.544 | 0.461 | 0.978 |
|  | TCC | 0.024 | 0.036, 0.012 | 4.554 | 0.033 | 0.115 |
|  | GCC | 0.013 | 0.013, 0.013 | 0.009 | 0.926 | 1.000 |

^1^p value calculated using permutation test and a total of 1,000 permutations

*Supplementary Table 12*. Epistatic interactions between genes in dyslipidemic HD patients (cases) and non-dyslipidemic HD subjects (controls)

|  |  |  |  |  |  |  | **FDR-adjusted** |
| --- | --- | --- | --- | --- | --- | --- | --- |
| **CHR1** | **SNP1** | **CHR2** | **SNP2** | **OR_INT** | **STAT** | **P** | **p-values** |
| 3 | rs7652589 | 4 | rs7041 | 0.837 | 0.624 | 0.430 | 0.990 |
| 3 | rs7652589 | 4 | rs1155563 | 0.997 | 0.0002 | 0.990 | 0.990 |
| 3 | rs7652589 | 4 | rs2298849 | 1.161 | 0.325 | 0.569 | 0.990 |
| 3 | rs7652589 | 9 | rs10881578 | 0.918 | 0.115 | 0.735 | 0.990 |
| 3 | rs7652589 | 9 | rs10776909 | 1.017 | 0.003 | 0.953 | 0.990 |
| 3 | rs7652589 | 9 | rs749759 | 1.110 | 0.176 | 0.675 | 0.990 |
| 3 | rs7652589 | 12 | rs1544410 | 0.936 | 0.082 | 0.775 | 0.990 |
| 3 | rs7652589 | 12 | rs2228570 | 1.175 | 0.432 | 0.511 | 0.990 |
| 4 | rs7041 | 4 | rs1155563 | 0.972 | 0.018 | 0.894 | 0.990 |
| 4 | rs7041 | 4 | rs2298849 | 1.686 | 3.348 | 0.067 | 0.484 |
| 4 | rs7041 | 9 | rs10881578 | 1.293 | 1.145 | 0.285 | 0.854 |
| 4 | rs7041 | 9 | rs10776909 | 1.014 | 0.002 | 0.960 | 0.990 |
| 4 | rs7041 | 9 | rs749759 | 0.956 | 0.034 | 0.853 | 0.990 |
| 4 | rs7041 | 12 | rs1544410 | 0.943 | 0.077 | 0.781 | 0.990 |
| 4 | rs7041 | 12 | rs2228570 | 0.960 | 0.036 | 0.850 | 0.990 |
| 4 | rs1155563 | 4 | rs2298849 | 1.285 | 0.837 | 0.360 | 0.990 |
| 4 | rs1155563 | 9 | rs10881578 | 0.859 | 0.470 | 0.493 | 0.990 |
| 4 | rs1155563 | 9 | rs10776909 | 0.750 | 1.335 | 0.248 | 0.854 |
| 4 | rs1155563 | 9 | rs749759 | 0.729 | 1.936 | 0.164 | 0.672 |
| 4 | rs1155563 | 12 | rs1544410 | 0.996 | 0.0003 | 0.986 | 0.990 |
| 4 | rs1155563 | 12 | rs2228570 | 1.325 | 1.900 | 0.168 | 0.672 |
| 4 | rs2298849 | 9 | rs10881578 | 0.850 | 0.340 | 0.560 | 0.990 |
| 4 | rs2298849 | 9 | rs10776909 | 0.574 | 2.723 | 0.099 | 0.580 |
| 4 | rs2298849 | 9 | rs749759 | 0.844 | 0.335 | 0.563 | 0.990 |
| 4 | rs2298849 | 12 | rs1544410 | 1.348 | 1.168 | 0.280 | 0.854 |
| 4 | rs2298849 | 12 | rs2228570 | 0.884 | 0.189 | 0.664 | 0.990 |
| 9 | rs10881578 | 9 | rs10776909 | 0.646 | 2.515 | 0.113 | 0.580 |
| 9 | rs10881578 | 9 | rs749759 | 0.810 | 0.748 | 0.387 | 0.990 |
| 9 | rs10881578 | 12 | rs1544410 | 1.056 | 0.053 | 0.818 | 0.990 |
| 9 | rs10881578 | 12 | rs2228570 | 1.099 | 0.145 | 0.703 | 0.990 |
| 9 | rs10776909 | 9 | rs749759 | 1.045 | 0.029 | 0.866 | 0.990 |
| 9 | rs10776909 | 12 | rs1544410 | 0.570 | 4.045 | 0.044 | 0.399 |
| 9 | rs10776909 | 12 | rs2228570 | 1.814 | 4.810 | 0.028 | 0.339 |
| 9 | rs749759 | 12 | rs1544410 | 0.946 | 0.050 | 0.823 | 0.990 |
| 9 | rs749759 | 12 | rs2228570 | 1.860 | 6.131 | 0.013 | 0.239 |
| **12** | **rs1544410** | **12** | **rs2228570** | **0.430** | **11.34** | **0.0006** | **0.027** |

| CHR1 Chromosome of first SNP |
| --- |
| SNP1 Identifier for first SNP |
| CHR2 Chromosome of second SNP |
| SNP2 Identifier for second SNP |
| OR_INT Odds ratio for interaction |
| STAT Chi-square statistic, 1df |
| P Asymptotic p-value |

*Supplementary Figure 1.* Cardiovascular mortality in hemodialysis patients in respect to *GC* rs2298849 polymorphism

Log rank test P = 0.797

| *GC* rs2298849 TT vs CC + CT | \| Parameter estimate \| \| --- \| | \| Standard error \| \| --- \| | \| Chi-square test \| \| --- \| | \| P value \| \| --- \| | \| 95% Lower CL \| \| --- \| | \| 95% Upper CL \| \| --- \| | \| Hazard ratio \| \| --- \| | \| 95% Hazard Ratio Lower CL \| \| --- \| | \| 95% Hazard Ratio Upper CL \| \| --- \| |
| --- | --- | --- | --- | --- | --- | --- | --- | --- | --- | --- | --- | --- | --- | --- | --- | --- | --- | --- |
|  | -0.020 | 0.077 | 0.067 | 0.796 | -0.172 | 0.132 | 0.961 | 0.709 | 1.301 |

*Supplementary Figure 2.* Infection-related mortality in hemodialysis patients in respect to *GC* rs2298849 polymorphism

Log rank test P = 0.061

| *GC* rs2298849 TT vs CC + CT | \| Parameter estimate \| \| --- \| | \| Standard error \| \| --- \| | \| Chi-square test \| \| --- \| | \| P value \| \| --- \| | \| 95% Lower CL \| \| --- \| | \| 95% Upper CL \| \| --- \| | \| Hazard ratio \| \| --- \| | \| 95% Hazard Ratio Lower CL \| \| --- \| | \| 95% Hazard Ratio Upper CL \| \| --- \| |
| --- | --- | --- | --- | --- | --- | --- | --- | --- | --- | --- | --- | --- | --- | --- | --- | --- | --- | --- |
|  | -0.387 | 0.188 | 4.229 | 0.040 | -0.756 | -0.018 | 0.461 | 0.220 | 0.964 |

*Supplementary Figure 3.* Neoplasm-related mortality in hemodialysis patients in respect to *GC* rs2298849 polymorphism

Log rank test P = 0.310

| *GC* rs2298849 TT vs CC + CT | \| Parameter estimate \| \| --- \| | \| Standard error \| \| --- \| | \| Chi-square test \| \| --- \| | \| P value \| \| --- \| | \| 95% Lower CL \| \| --- \| | \| 95% Upper CL \| \| --- \| | \| Hazard ratio \| \| --- \| | \| 95% Hazard Ratio Lower CL \| \| --- \| | \| 95% Hazard Ratio Upper CL \| \| --- \| |
| --- | --- | --- | --- | --- | --- | --- | --- | --- | --- | --- | --- | --- | --- | --- | --- | --- | --- | --- |
|  | -0.225 | 0.213 | 1.115 | 0.291 | -0.641 | 0.192 | 0.638 | 0.277 | 1.469 |

*Supplementary Figure 4.* The probability of survival in 70 hemodialysis patients in respect to circulating 25(OH)D at the start of the prospective study

Log rank test P = 0.112

| Vitamin D status < median vs  ≥ median | \| Parameter estimate \| \| --- \| | \| Standard error \| \| --- \| | \| Chi-square test \| \| --- \| | \| P value \| \| --- \| | \| 95% Lower CL \| \| --- \| | \| 95% Upper CL \| \| --- \| | \| Hazard ratio \| \| --- \| | \| 95% Hazard Ratio Lower CL \| \| --- \| | \| 95% Hazard Ratio Upper CL \| \| --- \| |
| --- | --- | --- | --- | --- | --- | --- | --- | --- | --- | --- | --- | --- | --- | --- | --- | --- | --- | --- |
|  | 0.290 | 0.185 | 2.465 | 0.116 | -0.072 | 0.653 | 1.787 | 0.866 | 3.689 |
